# Recruiting and retaining participants in e-Delphi surveys for core outcome set development: Evaluating the COMiT'ID study

**DOI:** 10.1371/journal.pone.0201378

**Published:** 2018-07-30

**Authors:** Deborah Ann Hall, Harriet Smith, Eithne Heffernan, Kathryn Fackrell, for the Core Outcome Measures in Tinnitus International Delphi (COMiT’ID) Research Steering Group

**Affiliations:** 1 NIHR Nottingham Biomedical Research Centre, Nottingham, United Kingdom; 2 Hearing Sciences, Division of Clinical Neuroscience, School of Medicine, University of Nottingham, Nottingham, United Kingdom; 3 Nottingham University Hospitals NHS Trust, Queens Medical Centre, Nottingham, United Kingdom; University of Liverpool, UNITED KINGDOM

## Abstract

**Background:**

A Core Outcome Set (COS) is an agreed list of outcomes that are measured and reported in all clinical trials for a particular health condition. An ‘e-Delphi’ is an increasingly popular method for developing a COS whereby stakeholders are consulted via a multi-round online survey to reach agreement regarding the most important outcomes. Many COS studies seek diverse, international input that includes professionals and healthcare users. However, the recruitment and retention of participants can be deterred by various factors (e.g. language barriers and iterative, time-consuming rounds). This report evaluates the effectiveness of recruitment and retention methods used in the Core Outcome Measures in Tinnitus International Delphi (COMiT’ID) study using participant feedback from healthcare users, healthcare practitioners, researchers, commercial representatives and funders.

**Methods:**

A range of methods were applied to recruit participants to the study and maintain engagement over the three rounds. Feedback on recruitment and retention methods was collected using a twenty-item online questionnaire, with free text comments.

**Results:**

A personalised email invitation was the most frequent recruitment route, and 719 professionals and healthcare users consented to take part. Retention of each stakeholder group ranged from 76 to 91% completing all three e-Delphi rounds. Feedback was given by 379 respondents. A majority of respondents were satisfied with the study methods that were implemented to promote retention. Over 55% indicated that their overall experience closely matched their expectations at the start of the study, and over 90% felt that their contribution was appreciated.

**Conclusions:**

This report highlights study methods that worked well with respect to recruitment and retention, and those that did not. Findings provide a unique contribution to the growing evidence base of good practice in COS development by demonstrating the relative effectiveness of recruitment and retention methods for an e-Delphi survey.

**Trial registration:**

This project was registered (November 2014) in the database of the Core Outcome Measures in Effectiveness Trials (COMET) initiative. The protocol is published in *Trials* (doi:10.1186/s13063-017-2123-0).

## Background

There is a need to standardise outcomes in order to enhance knowledge and improve design and reporting of clinical trials. A core outcome set establishes standards for outcome selection and reporting that should be measured and reported in every clinical trial of interventions for a particular health condition. An outcome can be viewed in two parts. First, the outcome domain refers to any aspect of that condition which matters most to patients and clinicians, such as tinnitus intrusiveness, sense of control, or impact on work. Second, the outcome instrument refers to how that domain is to be measured. Throughout this article, the term “outcome” refers to the general construct which includes the two concepts of what and how to measure, while the term “outcome domain” or “domain” is restricted to the concept of what to measure. A small number of agreed critically important outcome domains—known as a Core Outcome Set (COS)—forms a minimum set of outcome domains that should be measured and reported in every clinical trial, thus enabling results to be easily combined and compared [1, 2]. For flexibility, a COS does not necessitate that all outcome domains in a particular trial should be restricted to only those recommended, additional outcome domains can be collected and explored as well.

Various methods have been used in COS development and there is insufficient evidence to determine which is the most appropriate or efficient [2]. However, Delphi survey methods are one of the most frequent approaches used to achieve consensus among key experts in the field of interest (stakeholders) [2]. The Delphi survey uses iterative rounds of questionnaires listing candidate outcome domains and asking for personal ratings of each domain’s importance. Responses for each outcome domain are summarised across the stakeholder group and fed back anonymously within the subsequent round. Participants are able to consider the views of others before re-rating each item and can, therefore, change their initial responses based on the feedback from the previous rounds. One advantage of the Delphi method is that the feedback provides a mechanism for reconciling different opinions of stakeholders, and as such avoids the effect of dominant individuals and helps to minimise the influence of power differentials between different stakeholders that can occur with direct communication between participants [3, 4].

Electronic Delphi (‘e-Delphi’) surveys can led to increased sample size and diversity across international borders, reduced administration costs and time investments, and reduced managerial burden through digital data collection, management of individual anonymised responses and innovative participant communication through system-automated email reminders that were linked to individual data collection records indicating those who still need to complete the survey [5]. Additional advantages also include reliable technological support, robust data analysis and data sharing options, automated attrition monitoring and response tracking, and high levels of data security [6].

Given the reasons listed above, it is therefore no surprise that COS developers are increasingly relying on e-Delphi methods to reduce costs, increase participant targets and broaden their research outreach beyond national borders. Nevertheless, for e-Delphi survey participants, language barriers and the iterative, time-consuming rounds and lack of face-to-face interaction could be a deterrent to recruitment and retention. The time required to complete several rounds can be further complicated by long waits between rounds which might diminish interest and increase frustration [5]. Retention rates throughout the iterative e-Delphi process to the final round have not been reported consistently in the literature [4] but, for those that do, the numbers of participants recruited and retained remain highly variable from study to study. For example, the size of several recent international e-Delphi surveys recruiting multi-stakeholder groups that include healthcare users (members of the public) can range from 39 to 838 participants completing the final round, with retention rates of 19.5 to 87.1% [7–15]. Small group size and high attrition rates can impact on the final results. Small group size could lead to a COS that does not represent all the important outcomes associated with the condition and in turn this could impact on the endorsement and uptake of the COS in the larger community. Response bias could be introduced at to the level of consensus if the sample does not adequately represent each stakeholder group [2].

E-Delphi surveys used for COS development tend not to report in detail study recruitment and retention methods, nor to evaluate their effectiveness [4, 7–15]. A series of workshops engaged COS developers to explore what principles, methods and strategies they might want to consider when seeking healthcare user input into the development of a COS [3]. When identifying healthcare users as potential participants, it was recommended to gain a diversity of perspectives such as by promoting the study in health clinics, patient organisations, and via social media. With respect to promoting retention over time, recommendations to maintain interest over time from the workshops included managing expectations from the outset about timescales and keeping participants informed of progress, attending to common courtesies (e.g. building rapport with patients, showing appreciation for their contributions), building a sense of curiosity and excitement about COS development, and building a sense of ownership of the process [3]. Selecting motivated participants, as determined through an initial scoping survey, can also reduce potential attrition over iterative rounds [16]. Personally acknowledging, in the publication, only those participants who completed the entire Delphi process is another incentive to promote retention [17]. The Core Outcome Measures in Effectiveness Trials (COMET) handbook v1.0 [2] shares other suggestions for good practice such as personalised emails from a distinguished researcher in the field, and extending the closing date for a round.

E-Delphi experiences in other spheres can also be informative. Hsu and Sandford [18] recommended seeking endorsement from influential individuals, and personalising correspondence and reminders sent to participants. Experiences gained in a case study of a three-stage e-Delphi in the realm of natural resource and environmental management generated a set of practical advice for e-Delphi application, highly relevant to other contexts [6]. For recruitment and retention of participants, Cole and colleagues [6] recommended centralised communication through a dedicated website for sharing information about the research to legitimise the project and serve as a portal for communication between the researchers, participants, and other stakeholders. The authors also recommended using Internet-based databases and electronic mailing lists for recruiting potential participants.

While suggestions abound for enhancing recruitment and retention, to our knowledge, no previous e-Delphi studies have systematically evaluated and reported on the effectiveness of study recruitment and retention methods from the participant perspective. The handbook developed by the COMET Initiative [2] reiterates the uncertainty about how best to engage with healthcare users alongside other stakeholders in identifying what outcome domains to measure in clinical trials, and its authors call for further research to address this knowledge gap [2].

### Aim

This article reports and evaluates study design features that sought to address specific issues with recruitment and retention of healthcare users and professional participants in an e-Delphi survey. The evidence presented in this article responds to the call made by the COMET initiative for evidence-based COS development to inform good methodological practice in this area [2]. This work contributes to the growing evidence base of good practice in COS development.

## Methods

This article is based on the Study Management Team’s experience in the COMiT’ID study and reports on responses to a feedback questionnaire of the participant experience following three independent e-Delphi international surveys. COMiT’ID stands for ‘Core Outcome Measures in Tinnitus–International Delphi’ [19–21]. The project aimed to develop three separate COSs for tinnitus: one for sound-based interventions; one for psychology-based intervention; and one for pharmacology-based interventions. For each intervention, a modified e-Delphi survey was used to gain consensus on the same long list of outcome domains. To gain consensus across key stakeholders in tinnitus, we sought to recruit healthcare users, healthcare professionals, researchers, commercial representatives and funders, internationally, with a minimum target of at least 260 participants across three independent e-Delphi surveys [21]. Over three sequential rounds, each e-Delphi survey sought to achieve convergence of opinion from the different stakeholder groups about what outcome domains are critically important when deciding if the corresponding type of intervention is working [19–21].

The COMIT’ID study was reviewed and approved by three separate bodies; University of Nottingham Sponsor, NHS West Midlands—Solihull Research Ethics review board (REC reference: 17/WM/0095) and the Health Research Authority (HRA) before the study began, which is the requirement for all studies of nature in the UK. All e-Delphi survey participants gave informed consent using an online interface.

**Fig 1:**
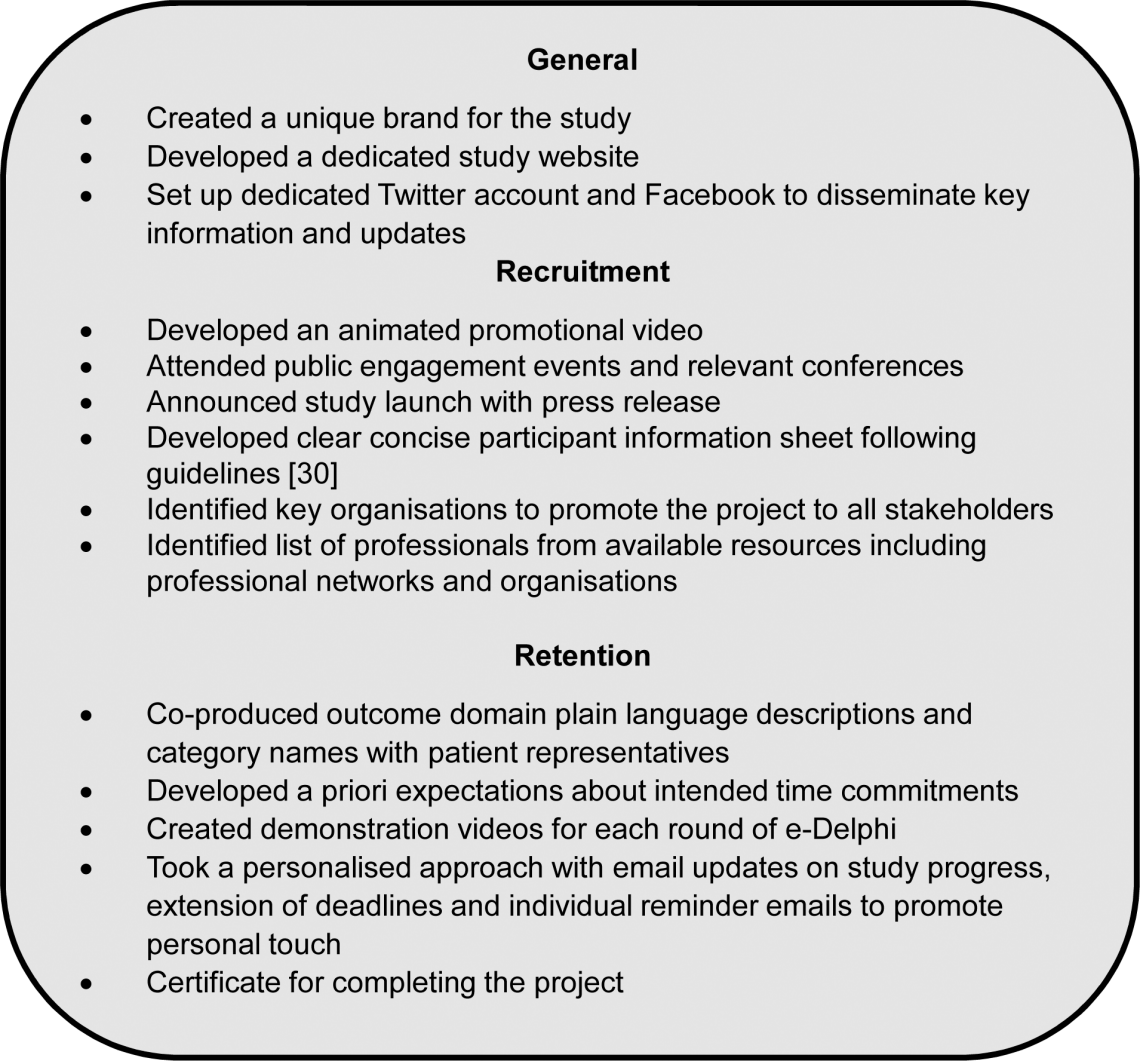
Summary of key recruitment and retention strategies.

In the set-up for the COMiT’ID study, the Study Management Team discussed with COS developers from other institutions their experiences in using social media to promote participation in an e-Delphi survey, as well as to devise retention strategies. Two key insights were to i) use YouTube videos for recruitment, and ii) update all stakeholders on progress at regular points in the process. Discussions inspired the Study Management team to develop diverse methods for promoting recruitment and retention throughout the project. Details of the methods are summarised in the following sub-sections (see also Fig 1). To improve the appeal and keep healthcare users fully engaged in the study, the two healthcare users with lived experience of tinnitus, referred to as Public Research Partners, commented on the feasibility of the e-Delphi survey design, and reviewed study documentation (advertisements, Information Sheets, YouTube video demonstrations for the survey).

### General promotional and recruitment methods

The COMiT’ID study created a unique brand identity, with its own acronym and logo, which was used in all documentation and advertising. General routes to promoting the COMiT’ID study and providing updates primarily used e-channels as these were cost-effective and reached an international audience.

A dedicated COMiT’ID study webpage was created, and hosted on the website of the lead organisation to provide an overview of the study, who should take part and what is involved, and information on the study team (webpage is no longer active). A dedicated Twitter account (@COMITIDStudy) and the lead organisation’s Facebook page (hearingnihr) were used to disseminate key information about the study including progress updates, participant information, recruitment announcements, COMiT’ID study articles and occasionally to answer queries from potential or existing participants. To broaden access, all features contained a hyperlink to the different communication channels.

An animated video hosted on the lead organisation’s YouTube channel [22], which provided a visual overview of the project and called for members of the public and professionals to register. Additionally, the COMiT’ID team attended public engagement events and relevant national and international conferences [22–28]. At these events, the video was showcased, and postcards with the slogan “I’m supporting the COMiT’ID study” were used to promote the study and to provide photo opportunities with influential individuals and professional colleagues to endorse the study. A press release from the University of Nottingham calling for research participants [29] led to a broadcast on a regional news programme (BBC East Midlands Today). All promotional information conveyed straightforward messages to the public focussing on their chance to “tell researchers what matters to you”, “have your say and influence future research”, and that “your opinion will help guide the research of tomorrow”.

Participant information sheets were designed in line with the recommendations from Knapp et al. [30] and with input from two Public Research Partners [21]. Plain English explanations of the study aims, process, and benefits were provided in clearly marked subsections. Separate participant information sheets were created for professionals and healthcare users. This ensured the information sheets were concise and appropriate for the intended audience. To emphasise the importance and appeal of the study, a brief overview of the study was created for the front page. Participants were made aware of the longer-term benefits the research would have on tinnitus management.

### Recruitment methods for healthcare users

Healthcare users were targeted using conventional methods of advertising for a clinical research study including poster advertisements in clinics in Primary Care West England and Audiology clinics (lead organisation and five approved Participant Identification Centres), in the UK and internationally (Germany, France, Portugal, Italy, Sweden, Mexico and Brazil) with COMiT’ID study “advocates” assisting with the local recruitment of healthcare users. Email invitations were circulated to all members of the British Tinnitus Association and the NIHR Nottingham Biomedical Research Centre participant database, which collectively reach thousands of people experiencing tinnitus. Newsletter articles created by the study team were shared via key communication channels, including articles and blogs for several patient organisations in UK, France and Germany (e.g. [31]). A ‘research news’ feature was created for a global online tinnitus support forum [32] to recruit healthcare users.

### Recruitment methods for professionals

A number of resources were used to produce a comprehensive list of professionals with relevant expertise to send personalised email invitations for each e-Delphi survey. These resources included (i) relevant conference proceedings from last 3 years for presenting authors, (ii) corresponding authors of tinnitus clinical trials identified in a systematic review [33], (iii) tinnitus systematic review authors (Cochrane or otherwise) from preceding 5 years, and (ii) editors of relevant scholarly journals. Commercial participants were also identified via direct contact with the relevant Research Consortium (representing medical device and pharmaceutical sectors).

All those identified on the list were emailed a personalised invitation to participate in the study from lead members of the COMiT’ID team. All professionals were encouraged to share the invitation with any colleagues having relevant expertise. An email invitation was also circulated to all members of relevant professional networks and organisations with tens of thousands of professionals across the globe (e.g. the British Society of Audiology, British Academy of Audiology, the TINNET network funded by an EU COST Action BM1306, ENT and Audiology News, the International Collegium of Rehabilitative Audiology, and the Pharmacological Interventions for Hearing Loss Working Group at the Hearing Centre of Excellence in the USA).

### General retention methods

Keeping participants fully engaged once recruited so that there is a low attrition rate is one of the challenges of conducting an online three-round Delphi survey [18]. A variety of methods were applied to retain participants throughout the e-Delphi process, through updates on social media, video demonstrations and personalised reminder emails.

The e-Delphi surveys were managed using DelphiManager software maintained by the COMET initiative (University of Liverpool: http://www.comet-initiative.org/). Details of the e-Delphi survey are reported in the study protocol [21]. Each e-Delphi survey started with a list of 66 outcomes with plain language descriptions, and grouped into categories. A number of steps were taken to ensure clarity of descriptions [21; 34]. Headings were ordered alphabetically, and within those, outcomes were also arranged alphabetically. To minimise any barriers to comprehension, the list of outcomes and their associated plain language descriptions were co-produced with two Public Research Partners and a Patient and Public Involvement Manager. This ensured that the outcomes were described so that all participants, including healthcare users, interpreted the meanings clearly and consistently across stakeholder groups. Independent lay reviews of these definitions were carried out by 14 members of the British Tinnitus Association’s Users’ panel, all with lived experience of tinnitus and experience of lay reviewing, and five clinical experts from the British Tinnitus Association Professional Advisory Committee, with subsequent modification to promote readability across a wider audience [21].

**Fig 2:**
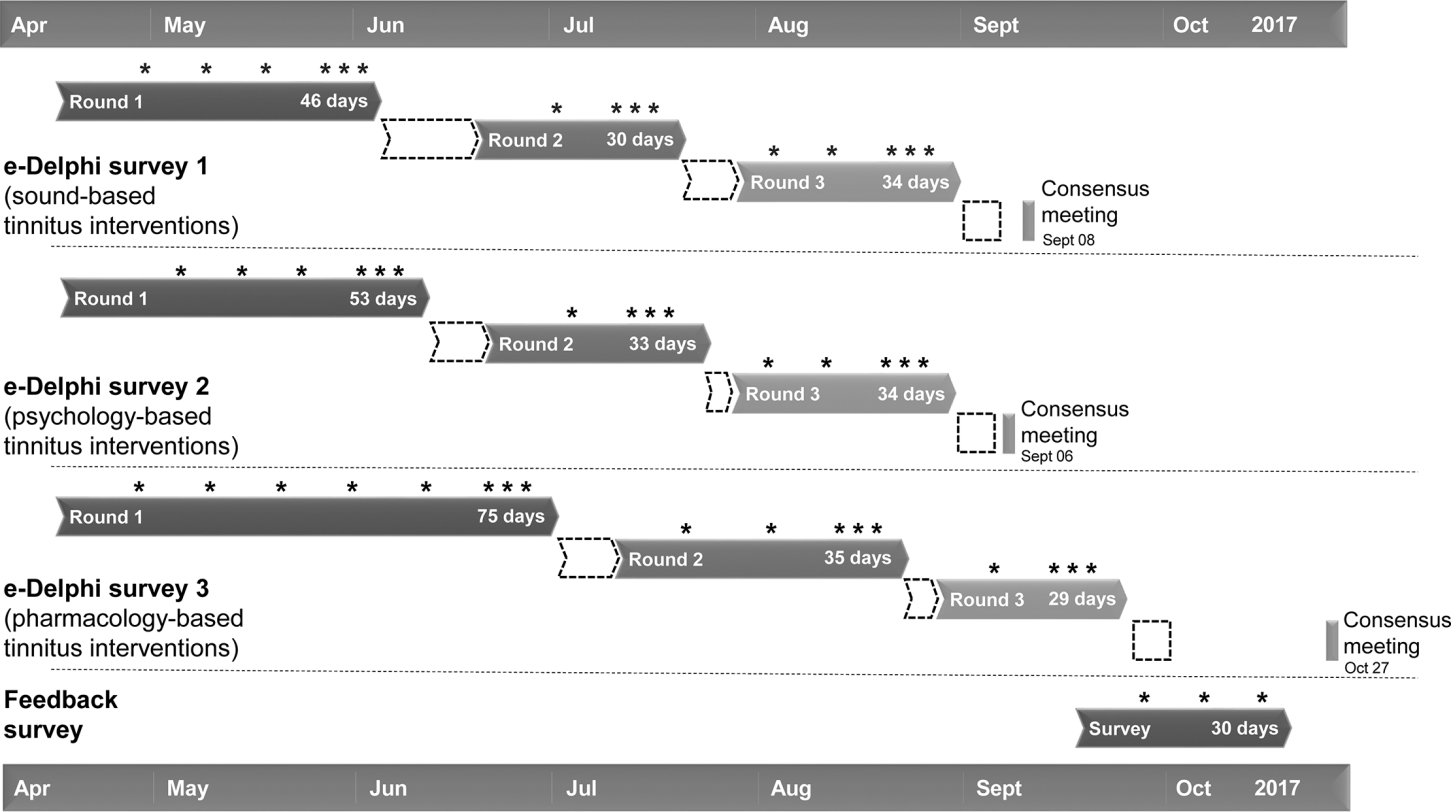
Schematic timeline of the three e-Delphi surveys and the feedback questionnaire. Solid fill rectangles denotes when the survey was open, with exact dates given. White fill rectangles denote when the 1–9 scores were being analysed. Asterisks indicate the date of the survey reminder emails to participants who had not completed or fully completed the survey at that time.

Each e-Delphi survey involved three rounds of rating the list of outcomes. Although the individual time taken to complete each round, the duration of each round and the time period between rounds were variable, clear *a priori* expectations about intended time commitments were established. For example, each round was estimated to be open to participants for about 45 days, subsequent rounds were estimated to be open within one week of the preceding round closing. A mean of 41 days (SD = 14.9) and 8.5 days (SD = 4.4) was achieved, respectively (see Fig 2).

### Demonstration videos

For each round, a short demonstration video was developed to supplement the written instructions particularly in giving participants a visual step-by-step guide on how to complete the online survey round. The videos are hosted on the lead organisation’s YouTube (see [35]) and could also be accessed via a hyperlink within the introductory page of each survey round. As examples, the videos for rounds 2 and 3 are available as Supplementary files (“S1 Appendix” and “S2 Appendix”, respectively). The videos were created using a screen-capturing tool that recorded the survey demonstrations on the computer (Camtasia Studio, TechSmith, Michigan, US). The demonstrations included annotations to highlight important elements of the survey and an audio explanation of the required steps. A clear explanation of the DelphiManager software was provided with clear instructions on the layout of each question page (outcome domain names, descriptions, rating scale and progress bar), how to provide optional feedback on each domain, and how to save and exit. The meaning of the 9-point response scale was re-iterated and respondents were instructed regarding how to interpret the feedback being provided. Importantly, participants were reminded to remember their stakeholder group affiliation and thanked for their contribution to the study.

### Keeping in touch / reminders

To maintain respondent’s engagement in the completing each round and between rounds, particularly over the summer holiday period, a variety of reminder methods were used. To build rapport with all respondents, email and social media updates about study progress were sent throughout the process, including announcements of the date to expect the next round of the survey so participants were not be caught unaware. Throughout the study, participants were thanked for the contribution to the study. Reminder emails were used to encourage participants to complete each round of the survey, and emphasise that their views matter and that for the results to be meaningful, it was important to complete the whole e-Delphi process. If participants were unable to complete the survey round by the deadline, deadlines were extended, when possible, to accommodate participants’ schedules. Personalised reminder emails were sent to promote a personal link, and encourage professionals to meet the deadlines. Participants were informed of the number of participants who had completed the round so far in their stakeholder groups to inspire them to complete the round. On competing the e-Delphi survey process, each participant received a certificate thanking them for completing and for their commitment to the project.

### Feedback questionnaire

Recruitment numbers, retention rates, social media following, website visits and video views are all informative performance indicators giving proxy markers on the general effectiveness of recruitment and retention methods. In addition, a 20-item follow-up questionnaire was conducted to collect feedback regarding how people found out about the e-Delphi and about their perspectives on recruitment and retention methods (see “S3 Appendix”). The feedback questionnaire was created using SurveyMonkey (https://www.surveymonkey.com) and emailed to all e-Delphi participants 10 days after closing round 3 of each survey. Stakeholder groups spanned healthcare users (people with lived experience of tinnitus), healthcare practitioners, clinical researchers, commercial representatives and funders. Some individuals consented to participate in more than one e-Delphi survey, and so when those duplicates had been accounted for there were 641 unique individuals invited to complete the feedback questionnaire. Most questions required the respondent to provide feedback via a 5-point Likert scale (‘very satisfied’ to ‘very dissatisfied’), with the option to type further free text explanations. A mixed methods analysis calculated descriptive statistics of the response patterns and explored themes arising from the free text comments.

From those invitations, 379 responses were received (59.1% response rate). Respondents came from 31 countries across Europe, Northern America, Eastern Mediterranean, South-east Asia and Australasia, and were representative of the e-Delphi participants (see “S4 Appendix”). Many respondents had English as their first language (257/375, 69%), with this proportion being higher in the healthcare user group (199/220, 90.5%). Stakeholder representation was broadly representative of the e-Delphi survey. Just over half the respondents identified themselves as healthcare users (224/379, 59.1%), with the remainder being healthcare practitioners (87/379, 23.0%), researchers (41/379, 10.8%), and commercial representatives or funders (23/379, 6.1%). Over 90% of the respondents to the feedback questionnaire were those who had completed all three rounds (350/379, 92.3%). Four healthcare users completed the feedback questionnaire, but did not complete any e-Delphi rounds and so these were not analysed further. Different reasons were given for why they chose to withdraw (i.e. one did not want to complete the survey on their iPad, one had a family bereavement, and one found it too difficult to rate the domains).

## Results

We evaluated the effectiveness of the e-Delphi recruitment and retention methods using data from the feedback questionnaire. Selected free text responses describe both positive and negative effects from the respondents’ perspective. All anonymised data from the feedback questionnaire can be found in “S5 Appendix”.

### Indicators on the effectiveness of promotional and recruitment methods

The dedicated COMiT’ID study webpage was one of the primary methods for promotion. On the date the feedback questionnaire closed (14 October 2017), the webpage had received a total of 3,308 unique views, with the greatest activity in May and June 2017 and an average viewing time of 2 minutes 9 seconds. Overall 719 participants consented to take part in the three e-Delphi surveys, exceeding the initial upper target of 420 and appealing equally to healthcare users (n = 384) and professionals (n = 335).

To get an indication of the recruitment route for the e-Delphi surveys, the feedback questionnaire first asked how individuals found out about the COMiT’ID study (Table 1). The majority of respondents (155/375, 41.3%) reported that a personalised email invitation from the Nottingham team alerted them to the study. For professionals, personalised email invitations were particularly successful in reaching those based outside the UK. For healthcare users, an invitation that was mailed out to the Nottingham participant database was the most likely information source. Other successful routes to recruitment of healthcare users were the social media posts by TinnitusHub, newsletters by the British Tinnitus Association and the regional TV appearance. Twitter reached a very small number of respondents, perhaps because the @COMITIDStudy had less than 125 followers in the six months that it was launched prior to opening the e-Delphi survey. Website analytics revealed that, on the date the feedback questionnaire closed (14 October 2017), the promotional video had been watched 1,054 times.

**Table 1:** Recruitment methods by which participants found out about the COMiT’ID study.

Recruitment method	Stakeholder group								Unknown
	Healthcare users		Healthcare practitioners		Clinical researchers		Commercial representatives and funders		
	UK	Non-UK	UK	Non-UK	UK	Non-UK	UK	Non-UK	
**Participant identification centre**									
Clinician mentioned it to me	1	0	3	13	0	2	0	1	0
Poster in healthcare clinic	4	1	0	0	0	0	0	0	0
**COMiT’ID webpage**	5	0	1	1	1	0	0	0	0
**Social media**									
Twitter	4	1	0	0	0	0	0	0	0
Facebook	13	1	0	0	0	0	0	0	1
TinnitusHub: online support forum	8	24	0	1	0	0	0	0	0
TINnitus NETwork website	0	1	0	2	0	0	0	0	0
Other social media	3	0	0	0	0	0	0	0	0
**Newsletters**									
British Tinnitus Association	30	0	3	0	0	0	4	0	2
Other patient organisations	5	0	0	1	0	0	0	0	0
**Events and conferences**									
TV (BBC East Midlands Today)	18	0	0	0	0	0	0	0	0
ITS World Congress, Warsaw	0	1	0	1	0	1	0	0	0
TINnitus NETwork Meeting, Madrid	0	0	1	1	1	0	0	0	0
IFOS, Paris	0	1	0	0	0	0	0	0	0
**Personalised email invitation**	43	11	18	32	12	16	0	16	7
Other	8	4	0	6	0	1	1	1	1
Cannot remember	19	10	1	1	1	3	0	0	1
**Total**	**161**	**55**	**27**	**59**	**15**	**23**	**5**	**18**	**12**

BBC = British Broadcasting Corporation, IFOS = International Federation of Otorhinolaryngology Societies, ITS = International Tinnitus Seminars, TV = Television.

The majority were ‘very satisfied’ or ‘somewhat satisfied’ (307/375, 81.9%) with the study overview provided in the Participant Information Sheet. (Table 2). Free text data (n = 65) revealed many positive comments saying that it was informative and interesting. For example, one respondent stated that it was “Interesting enough to make me want to take part”. Some stated that it was easy to understand and that it made the purpose of the study clear. For example, one wrote: “[The] objectives and my role [were] clearly defined”. Only 12 respondents rated that they were dissatisfied with the overview, mostly being healthcare users (n = 11). An analysis of the free text comments showed that some respondents felt that the information sheet was too lengthy, and that the terminology was difficult to understand. For example, one wrote: “As a "sufferer" as opposed to an academic, I had some difficulty in getting my head round some of the concepts”. Some felt that the purpose and requirements of the study were unclear. For example, one said: “Initially it was unclear what you were expecting in terms of scoring "outcomes". Another thought the study would be about treating tinnitus: “[I] was hoping to get help out of this [study]”.

**Table 2:** Feedback questionnaire findings showing satisfaction with the study overview in the participant information sheet, split by per stakeholder group and first language.

Rating	Stakeholder group								Unknown
	Healthcare users		Healthcare practitioners		Clinical researchers		Commercial representatives and funders		
	English as first language	EAL	English as 1^st^ language	EAL	English as 1^st^ language	EAL	English as 1^st^ language	EAL	
Very satisfied	85	8	20	39	11	16	8	7	0
Somewhat satisfied	64	5	8	12	4	10	1	4	2
Neither satisfied nor dissatisfied	35	6	4	0	0	0	2	1	4
Somewhat dissatisfied	6	1	0	1	0	0	0	0	0
Very dissatisfied	4	0	0	0	0	0	0	0	0
I didn’t read it	4	1	0	2	0	0	0	0	0
**Total**	**198**	**21**	**32**	**54**	**15**	**26**	**11**	**12**	**6**

EAL = English as an additional language.

The majority of respondents were ‘very satisfied’ or ‘somewhat satisfied’ with the study overview provided in emails and online survey pages. The free text comments (n = 54) again showed that many respondents found these materials to be informative and easy to understand. One said that it was: “Clear and easy to follow and not too long”, whilst another said: “The information answered all of my questions about the study”. Only 17 were dissatisfied, mostly being healthcare users (n = 13). Analysis of all the free text comments revealed that some felt the information provided was somewhat difficult to understand and that the aims and requirements of the study were unclear. For example, one said: “It made the commitment sound more intensive than it actually was and may have put some people off”. There was no indication that English as a first language, or as an additional language, influenced satisfaction ratings.

### Indicators on the effectiveness of retention methods

Retention of participants throughout all three e-Delphi surveys ranged from 76 to 91% completing round 3, depending on the stakeholder group (Table 3). All those who contributed to round 3 had to have completed all preceding rounds, with a completion rate of at least 40% of items.

**Table 3:** Total number of participants who completed each round of the e-Delphi surveys.

Stakeholder group	Consented	Round 1	Round 2	Round 3	Retention%)
Healthcare user	384	358	305	272	76.0
Healthcare practitioner	193	178	157	144	80.9
Clinical researchers	95	91	86	83	91.2
Commercial representatives and funders	47	43	38	33	76.7

Retention is shown from round 1 to round 3, reported separately for each stakeholder group.

COS developers have suggested that managing expectations from the outset is an important way to promote retention [3]. Over half indicated that their overall experience closely matched their expectations at the start of the study (217/375, 58.9%). Table 4 reports responses regarding study based on stakeholder group. Findings highlighted that a high proportion of healthcare practitioners, clinical researchers, and commercial representatives and funders (72.4%, 80.5% and 69.6%, respectively) reported that the process closely matched their expectations. Analysis of the free text comments (n = 88) suggested that many respondents simply did not have any specific expectations at the outset of the study. One commented: “This was the first time I have taken part in a study. I didn't have any definite expectations”. Several reported that the study exceeded their expectations. For example, one respondent was: “Positively surprised…[the] survey, information and visual aids [were] very engaging”.

**Table 4:** Feedback questionnaire findings showing frequency of ratings for “meets expectations” split by stakeholder group.

Overall experience versus expectations	Stakeholder groups				
	Healthcare users	Healthcare practitioners	Clinical researchers	Commercial representatives and funders	Unknown
Closely matched my expectations	103	63	33	16	2
Quite different from what I expected	98	15	7	3	0
Prefer not to say	13	7	1	4	2
Missing data	6	2	0	0	0
**Total**	**220**	**87**	**41**	**23**	**4**

In contrast, a majority of healthcare users (98/220, 44.5%) reported that the process differed from their expectations. Analysis of two other questions in the feedback questionnaire did not reveal any relations between ‘not quite meeting expectations’ and ratings of dissatisfaction with the study overview either provided by the Participant Information Sheet or by the emails and online survey pages. Analysis of the free text comments (n = 88) suggested that the e-Delphi survey was more demanding, complicated, and repetitive than respondents were anticipating. One wrote: “There were too many questions, many of them quite similar to each other and not all of them applicable to a lay person”. Another wrote: “I did not like the format of rounds 2 and 3. My opinions were asked for in round 1—great. But then it seemed to me that in rounds 2 and 3, the question was: 'this is what other people have said—does it change your mind at all?' And my answer was 'No—why would it change my mind!' As a result I found rounds 2 and 3 rather pointless”. In addition, some felt that the survey was not personally relevant or that they could not share their personal experiences to the extent that they were expecting. One noted: “I thought I would be writing down my experiences and suggestions”.

### General retention methods

Across all respondents, the majority (262/375, 69.9%) reported satisfaction with the clarity of outcome domain description. For instance, one respondent said “I felt they had been well thought out”. Only a small minority were dissatisfied (52/375, 13.9%). Table 5 reports satisfaction ratings split by stakeholder group and whether or not English was the respondents’ first language. Having English as an additional language did not appear to influence dissatisfaction with outcome domain clarity. Almost all of the respondents who were dissatisfied (i.e. 48/52) were healthcare users. The free text comments (n = 84) indicated that many respondents found the outcome domains somewhat difficult to understand. They felt that the language was too “technical” or too “medical” and that some domains were repetitive or difficult to distinguish from one another. Some reported that it was difficult to provide scores for domains that were not relevant to their personal experiences. For example, one respondent wrote: “Some were very confusing and not relatable to me as a sufferer, maybe they made more sense to a medical person?” Another respondent wrote: “[I] had to continually consciously remind myself that just because I didn't suffer from a specific symptom, it would still be a [terrible] symptom to suffer from and I should therefore mark it highly in terms of wanting a treatment to address it”. A small number of respondents found the term ‘outcome domain’ itself difficult to understand. For example, one respondent commented: “Even the phrase, "outcome domain" is alien to me, so I was really confused”.

**Table 5:** Feedback questionnaire findings showing satisfaction with outcome domain description, split by per stakeholder group and first language.

Rating	Stakeholder groups								Unknown
	Healthcare users		Healthcare practitioners		Clinical researchers		Commercial representatives and funders		
	English as 1^st^ language	EAL	English as 1^st^ language	EAL	English as 1^st^ language	EAL	English as 1^st^ language	EAL	
Very satisfied	53	8	20	29	7	16	5	5	2
Somewhat satisfied	57	5	6	22	6	9	5	7	4
Neither satisfied nor dissatisfied	40	3	5	2	1	1	1	0	2
Somewhat dissatisfied	33	3	1	1	1	0	0	0	1
Very dissatisfied	11	1	0	0	0	0	0	0	0
Missing data	4	1	1	1	0	0	0	0	0
**Total**	**198**	**21**	**32**	**54**	**15**	**26**	**11**	**12**	**6**

EAL = English as an additional language.

When asked about the length of time for completing each round of the e-Delphi survey the majority of respondents reported that this again closely matched their expectations (224/375, 59.7%). Free text comments (n = 43) included assertions such as: “I was quite happy with the length of time needed to complete each round (we were told about this in advance anyway) and could always save responses and go back to the survey later, which I did on a couple of occasions”. Another wrote: “[I] wanted enough time to work on it without being pressed, the format allowed this comfortably”.

A number of respondents reported that the time to complete each round was shorter than they expected (74/375, 19.7%). A similar number felt that the time to complete each round of the e-Delphi was longer than they expected (70/375, 18.7%). For example, there were some who reported in the comments that the e-Delphi survey was too time consuming. One respondent said: “It was OK. But the questions were long winded…2 rounds are enough! I got bored by the third round!”

In terms of the length of time between each round of the e-Delphi survey, the overwhelming majority of respondents were satisfied with the allotted time (286/375, 76.3%). This is supported in the free text comments (n = 49), with one stating that: “I was travelling during [the] summer so the [timing] suited me and I managed to complete all by the deadlines”. In particular, respondents felt that there was enough time to reflect on their responses without forgetting the previous round. One wrote: “[It was] long enough to reconsider but not too long to forget”, whilst another commented that it: “gave me time to reflect on my answers and think about my illness”. A very small number (5/375, 1.3%) reported dissatisfaction, with some respondents commenting that the length of time between rounds was “too long”. The remaining respondents gave either neutral responses (‘Neither satisfied or dissatisfied’, 74/275, 19.7%) or did not respond to that question (10/375, 2.7%). It is important to note that this question may not have been interpreted equally by all respondents because the actual length of time between completing successive rounds could vary a lot from person to person (Fig 2). For example, for e-Delphi survey 3 (pharmacology-based tinnitus interventions) the shortest possible length of time was 3 days between the closure of round 1 and the launch of round 2. Yet, at least one respondent experienced a ten-week interval between these same rounds because he completed both rounds shortly following the date of their respective launches (Fig 2).

### Demonstration videos

The number of views of the online demonstration videos was high at the start, but then declined over successive rounds. On the date the feedback questionnaire closed (14 October 2017), the video for round 1 had had 646 views, while the video for round 2 had received 342 views, and round 3 had received 123 views. The survey results showed that half of responders were ‘very’ or ‘somewhat’ satisfied with the demonstration videos (195/375, 52.0%). Free text comments (n = 60) showed they were perceived to be clear, informative, and helpful: “[a] very good tutorial as [to] how to use the survey”, and “Make it easier to understand the expectations of [the] study”. Several respondents acknowledged that the video format gave a useful alternative option for conveying important study information. One said: “This was helpful to explain in voice mode rather than written, and was very clear and user friendly”. Another said: “Seemed shorter and more informative than laborious emails”.

A third of respondents reported that they did not watch the demonstration videos (125/375, 33.3%), whilst a minority were dissatisfied (16/375, 4.3%). Free text comments demonstrated that several respondents had not been aware that the videos existed or at least could not remember having watched them. A small number of respondents experienced technical problems that prevented them from watching the videos. Several respondents felt that the videos were unnecessary because they repeated information already provided in written form or because the respondents found the e-Delphi survey to be self-explanatory. According to one: “the text explanations were sufficient”. Some regarded the videos as helpful, but not essential, or as potentially helpful to others. One respondent commented that they: “Repeated [the] written information. I understand some people might have found it easier to understand in this form”. Another stated that they were: “A bit repetitive…but a comforting reminder”.

### Keeping in touch/reminders

When asked about their satisfaction with how the team kept them informed of progress from round to round, the majority of respondents (313/375, 83.5%) were satisfied. From the free text comments (n = 44), the majority made positive comments about the communications sent by the study team, including finding them to be helpful, informative and clear. For example, one respondent said: “The study team always replied promptly when I e-mailed for help. I had a clear idea of how the study was progressing”. A small minority were dissatisfied (3/375, 0.8%), with some respondents providing negative comments about the communications from the study team, including finding them to be uninformative or excessive. The remaining respondents were either neutral (51/375, 13.6%) or did not respond to this question (8/375, 2.1%).

Similarly, when respondents were asked about their satisfaction regarding the frequency of email reminders from the team, over three-quarters were satisfied (287/375, 76.5%). From the free text comments (n = 47) most respondents felt that the frequency of the reminder e-mails was appropriate. For instance, one respondent commented that there were: “Just enough without being intrusive”. Some said that the reminders were helpful in encouraging them to complete the e-Delphi survey. One wrote: “I did need prompts sometimes if I didn't respond straight away as emails can easily slip down the list”.

A minority were dissatisfied with the frequency of reminders (17/375, 4.5%), commenting that there were too many e-mails or that these e-mails were sent prematurely. One stated that: “There were too many reminders when there were weeks [left] to complete the survey, I felt hassled by them”. Of the remaining responders, a number were ‘Neither satisfied nor dissatisfied’ with the frequency of reminders (54/375, 14.4%), stated they did not receive any email reminders (10/375, 2.7%) or did not respond to this question (7/375, 1.9%).

As communication with participants might influence perceptions about personal contribution [3], respondents were asked to indicate whether or not they felt their contribution was appreciated. Analysis of the results showed that over 90% of respondents (342/375, 91.2%) felt that their contribution was appreciated, with free text comments (n = 56) supporting that contact with the study team made them feel their participation was valued. One commented: “The frequent reminders…show that you value each and every response”, whilst another wrote: “They made you feel it was important”. Other comments indicated that some respondents were hopeful that their contribution would make a difference to future research or improved patient wellbeing. For example, one said: “I thought once it was done we would be forgotten, so it is very nice to be asked an opinion and hope it moves to help sufferers and practitioners alike”. Of the remaining respondents, a small number reported their contribution was not appreciated (25/375, 6.7%) but there were few free text comments to reveal the reasons behind these.

### DelphiManager software

Although the design of the software interface was one aspect of the research over which the Study Management Team had little control, it is nevertheless likely to be an important contributing factor to retention. Several items in the feedback questionnaire asked about experiences using the DelphiManager software. Seven respondents did not complete these questions, giving a total of 368 responses. Respondents were first asked how they felt about the ease of use of the survey software. The majority were ‘very satisfied’ or ‘somewhat satisfied’ (307/368, 83.4%) (Table 6), with only 26 (7.1%) being dissatisfied. From the free text comments (n = 79), two recurring issues causing some degree of dissatisfaction were the small font size of the outcome domain names and descriptions, and the inability to go back and review previous answers: “It was impossible to go backwards and see your previous responses. And the text was quite small on the page”.

**Table 6:** Feedback questionnaire findings showing overall satisfaction with e-Delphi software based on multiple functionality options and scoring.

	Ease of use of the survey software	Usefulness of the option to ‘save and exit’ and then log back into the survey	Usability of the 1–9 scoring options	Interpreting the graphical display of results in Round 3
Very satisfied	204	191	174	188
Somewhat satisfied	103	39	111	93
Neither satisfied nor dissatisfied	35	37	52	55
Somewhat dissatisfied	19	2	25	11
Very dissatisfied	7	2	6	5
I didn't use this option	n/a	97	n/a	n/a
I didn’t complete round 3	n/a	n/a	n/a	16
Missing data	7	7	7	7
**Total**	**375**	**375**	**375**	**375**

In terms of the process of scoring, one question asked how respondents felt about the usability of the 1–9 response scale. Again, the majority were ‘very satisfied’ or ‘somewhat satisfied’ (285/368, 77.4%), with only 31 (8.4%) being dissatisfied. Free text comments (n = 84) revealed a preference for a smaller number of response options, even from some respondents who said they were satisfied. This view is captured by the quote: “I could easily differentiate between a very important (7–9) or a not-so-important (4–6) stuff, but I couldn't clearly determine if it was 7, 8 or 9”.

In round 3, feedback was given in the form of up to four coloured histogram charts [see “S2 Appendix”], one for each stakeholder group, and one question asked how respondents felt about interpreting these graphical displays. Most responses indicated some degree of satisfaction (281/368, 76.4%). Free text comments (n = 59) revealed that some respondents felt there was a benefit of seeing others’ responses in particular two researchers specifically highlighted the benefits of reflecting on the healthcare users’ perspective: “It seemed to me the most interesting thing of the survey ….!! Thanks to this information I have modified some of my answers taking into account the opinion of the patients”.

Finally, respondents rated how they felt about the option to ‘save and exit and then log back into the survey’. Again, the majority were ‘very satisfied’ or ‘somewhat satisfied’ (230/368, 62.5%). The free text comments (n = 52) indicated the reassurance that this gave. Examples include: “I felt more relaxed while doing the questionnaire—had a choice to stop if I needed to” and: “That was very useful if you needed to be called away for some reason. I didn't have to, but the option was there”. A substantial number (n = 97) reported that they did not use the save and exit option, with some explaining their preference to complete the survey in one session in the comments: “Given the issues accessing the survey, it was easier/less risky to simply complete it all in one go”, and “I didn't use the feature, as I felt it better for consistent responses if the survey was completed in one session”.

## Discussion

**Fig 3:**
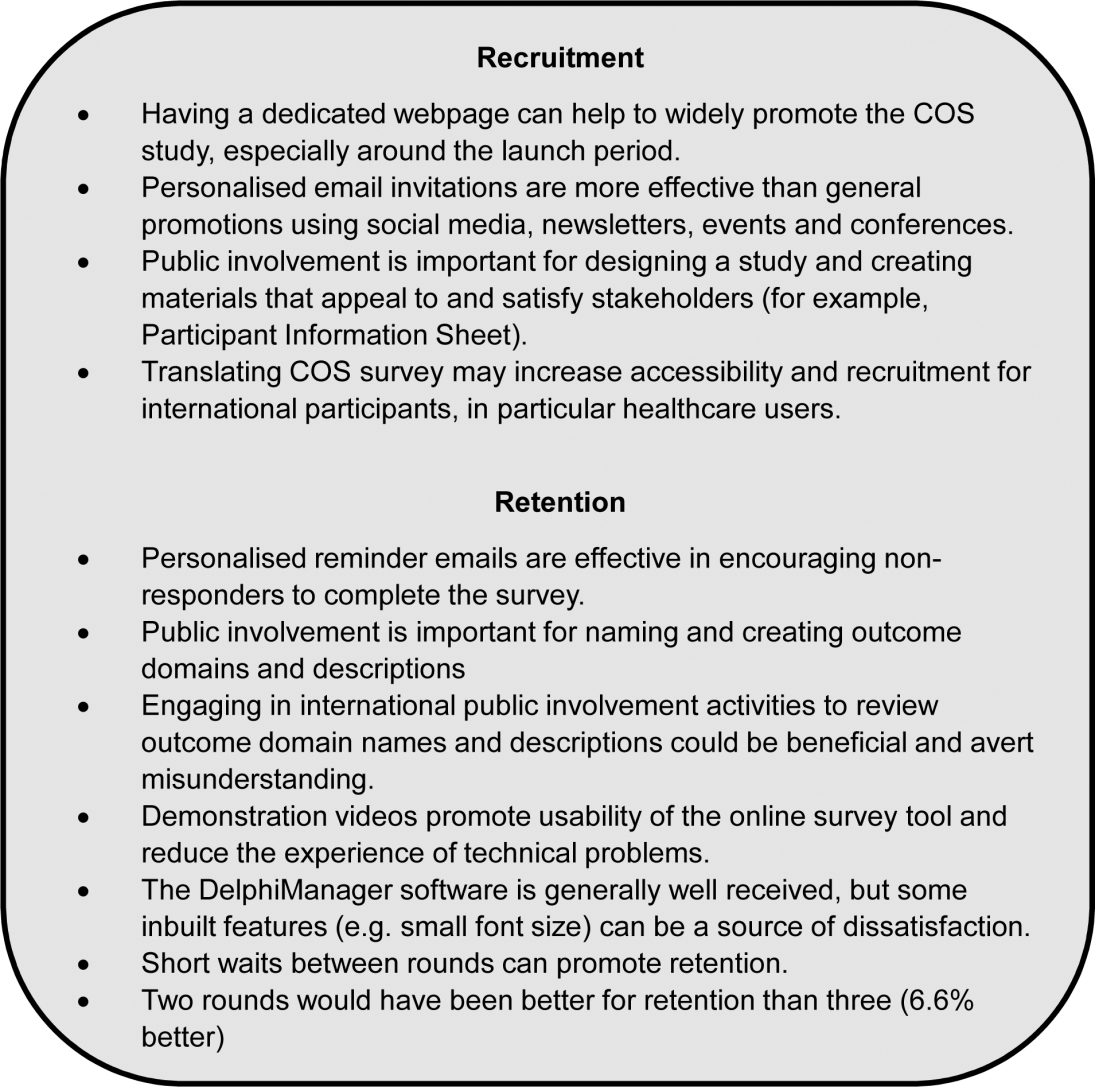
Recommendations for future COS developers. Main observations and lessons learned from the COMiT’ID study form a set of recommendations for others to consider.

Healthcare users are increasingly being included as participants in the COS decision-making process. For example, the COMET handbook v1.0 [2] noted that 19% (44/227) projects that had been published up to the end of December 2014, reported including healthcare users as participants, while this had grown to 88% (112/127) in COS projects that were ongoing as of 12 April 2016. The handbook authors also noted that the most commonly used consensus method was the Delphi technique (38% of studies) [2]. The COMET handbook v1 [2] encourages researchers to develop and disseminate advice regarding issues to consider when developing COS. With this in mind, COS developers in this growing field can learn valuable lessons from the recruitment and retention methods used and evaluated in the COMiT’ID study. Main observations and recommendations are summarised in Fig 3, and some of these points are discussed in more detail below.

A strength of this study was implementing the good practice recommendations from the COMET handbook v1 (see Section 10) [2] on how to enhance recruitment and retention, despite the COMiT’ID protocol being developed before its publication. Although recommendations are proposed in the context of healthcare users [2], they are equally relevant to professional participants.

### A critical self-reflection on recruitment methods

For the COMiT’ID study, personalised email invitations were the most effective method in terms of numbers of consented participants. This is also a common approach used in other studies which have high response rate [7–12;14;15]. This indicates that to be successful, COS developers need to access experts through the appropriate infrastructure including professional networks and patient organisations. Other methods also made high demands on staffing resource, but yielded relatively few consented participants in our study. These included posters placed in clinical settings, the dedicated COMiT’ID study webpage and Twitter account, and newsletter articles published by several patient organisations. The COMET handbook v1.0 [2] recommends that a range of sources are used to reach out to healthcare users. The COMiT’ID study experience would extend that recommendation to professionals for whom recruitment also required substantial planned resource. A variety of recruitment methods was advisable, tailored to reach each stakeholder group. Nevertheless, social media platforms are perhaps most effective only if they are already established rather than being set up specifically for the COS development project, or if the target audience are known to be typical social media users [36]. Although there is substantial interest in the use of social media as a component of an effective patient recruitment strategy, challenges regarding engagement, representativeness, obtaining buy-in, and resources required have been noted. For example, the COMET handbook v1 [2] is cautious about the value of accessing patient communities via social media since there is evidence of poor response rates through social media, and those who do participate may be of limited diversity due to self-selection [36].

As is so often the case [9;11;12;14], resource limitations prohibited translation of the COMiT’ID e-Delphi survey into multiple languages. Our experience indicated that restricting study materials to the English language did not seem to be a major barrier to international recruitment for professional stakeholders, but may have been for healthcare users. Issues relating to accessibility by international participants through translation of COS surveys warrants further attention. Indeed, many of the international COS studies reviewed in this article did not mention whether or not there were language restrictions [7;8;10;15], suggesting that this is perhaps a somewhat neglected topic. One COS study that did appear to translate the survey did not report details, but did raise a concern about the equivalence of the translated versions and the potential bias this could introduce [13]. We also note that the DelphiManager software used does not readily integrate data from multiple languages in the same survey.

### A critical self-reflection on retention methods

The COMET handbook v1 [2] recommends that COS developers incorporate the words that patients use to label and explain outcome items in a Delphi survey when creating the long list of outcome domains. While the handbook recommends qualitative methods, such as focus groups in which people describe their views and experiences in their own term, the COMiT’ID study advocated patient involvement through the two Public Research Partners who were members of the Research Steering Group (see also [7]). There is no evidence to choose which method might be preferable, but we believe that some form of input from people with lived experience of the condition is better than none. Nevertheless, despite involving two Public Research Partners in determining the wording of the outcome domains and their descriptions and despite involving numerous members of the British Tinnitus Association in piloting and refining those outcome labels and explanations [21;34], a substantial number of e-Delphi participants reported that they still found the language somewhat difficult to understand. These comments are much more likely to relate to a small number of the medical constructs (including ‘pharmacokinetics’, ‘neuroendocrine hormones’ and ‘oxidative stress’) than the majority of outcome domains which described everyday experiences (such as ‘concentration’, ‘anxiety’, and ‘impact on relationships’). We believe that those difficulties would have been even greater without such patient input since these constructs were subject to considerable study team discussion during set up. For COS development in diseases where there are many medical or technical outcomes to consider, this could be a challenge not only for participants but also for the study team. The study findings suggest that outcome domain names and their descriptions could have benefitted from a wider lay review with non-native English language speakers to improve accessibility by increasing understanding. This limitation could have been overcome by engaging the European professional members of the Research Steering Group to lead some of the patient involvement activities in their own countries, either in the form of workshops or an online survey. The Study Management Team did not act on the small number of negative participatory experiences that were received in the round 1 free text comments, due to resource limitations and time pressures for opening round 2. However, future work could consider a role for patient involvement here, especially where comments pertain to the understanding of specific outcome domain names and descriptions.

Two- and three-round Delphi surveys are common in COS development studies [2]. Here we chose a three-round Delphi in order to give participants the chance to first reflect on their peers’ viewpoint in round 2 before being invited to consider the viewpoint of their stakeholder group and that of the other stakeholder groups in round 3. We did this because we anticipated a diversity of opinions across tinnitus experts and so we expected that the process of reaching a consensus might require successive opportunities for participants to reflect on the candidate outcome domains. However, our findings of an attrition rate of 6.6% between rounds 2 and 3, and comments indicating that the value of round 3 was not recognised by all participants demonstrate the potential for a three-round Delphi survey to have negative implications for retention.

### Limitations of the study

The majority of respondents in the feedback questionnaire were European and North American, reflecting the geographical bias of the e-Delphi survey. This pattern is similar to other COS studies [8–12;14,15]. Other countries and non-English language speakers were under-represented, primarily because the Study Management team did not have the resources to target recruitment strategies outside Western countries (such as Africa), nor to translate the survey into non-English languages. The potential impact of this is not easily measurable but it may limit the generalisability of the study to all regions of the world. While our retention rates in the e-Delphi survey met the 80% threshold for each stakeholder group as recommended by [2], it is uncertain to what degree the participant feedback reported in this article is representative of the views of those who had chosen to drop out since only 29 of the 379 respondents to the feedback questionnaire had withdrawn from the e-Delphi survey at rounds 1 or 2, and we did not ask individual reasons for drop out. While it might be expected that those who completed round 3 were most likely to be the ones who were satisfied with the e-Delphi methods, some of the negative free text comments might help to inform reasons for withdrawal. These generally fell into two categories namely the demanding and repetitive nature of the survey rounds, and difficulty understanding the general concept of an outcome domain, or the names and plain language descriptions of individual outcome domains. Neither comment distinguished those who dropped out from those who completed round 3.

### Conclusions

The findings from this evaluation contribute to the growing evidence base of good practice in COS development, providing an informative case study to support future investigators in the planning of their study. Information regarding the relative effectiveness of recruitment and retention methods can help inform the allocation of time and cost to enable future COS studies to successfully achieve sample targets and ensure a positive participant experience.

## Supporting information

S1 Appendix Demonstration video. Demonstration for completing COMIT’ID online survey round 2.
(WMV)

S2 Appendix Demonstration video. Demonstration for completing COMIT’ID online survey round 3.
(WMV)

S3 Appendix 20-item feedback questionnaire. (DOCX)

S4 Appendix Questionnaire respondents. List of feedback questionnaire respondents and which country they came from, compared with participants in the e-Delphi surveys.
(DOCX)

S5 Appendix Anonymised dataset. (XLSX)

## Abbreviations

BBC: British Broadcasting Corporation
COMET: Core Outcome Measures in Effectiveness Trials
COMiT’ID: Core Outcome Measures in Tinnitus–International Delphi
COS: Core Outcome Set
COST: Cooperation in Science and Technology
EAL: English as an Additional Language
ENT: Ear, Nose and Throat
EU: European Union
IFOS: International Federation of Otorhinolaryngology Societies
ITS: International Tinnitus Seminars
TINNET: European TINnitus NETwork
TV: Television
UK: United Kingdom
USA: United States of America
