# Supplementary material for: Recruiting and retaining participants in e-Delphi surveys for core outcome set development: Evaluating the COMiT'ID study
Source: PLoS One. 2018 Jul 30;13(7):e0201378. doi: 10.1371/journal.pone.0201378 (PMC6066228; doi:10.1371/journal.pone.0201378)
Supplement: S3 Appendix — (DOCX) [file pone.0201378.s003.docx]

**Feedback survey**

In this short feedback questionnaire (20 questions), the study team would be grateful to hear about your experiences of being a participant. This will help us to improve the design of any similar online surveys in the future.

1. How did you find out about the study?

• Email invitation directly from the Nottingham team

• Twitter

• Facebook

• Other social media

• Poster in healthcare clinic

• Clinician mentioned it to me

• NIHR Nottingham Biomedical Research Centre website

• TinnitusTalk

• EU COST TINNET website

• British Tinnitus Association

• Other patient organisation (e.g. German Tinnitus Association)

• BBC East Midlands Today news feature

• Can’t remember

• Other ______

2. How did you feel about the study overview provided in the Information Sheet?

• Very satisfied

• Somewhat satisfied

• Neither satisfied nor dissatisfied

• Somewhat dissatisfied

• Very dissatisfied

• I didn’t read it

3. How did you feel about the study overview provided in the emails and online survey pages?

• Very satisfied

• Somewhat satisfied

• Neither satisfied nor dissatisfied

• Somewhat dissatisfied

• Very dissatisfied

• I didn’t read them

4. How did you feel about the helpfulness of the demonstration videos?

• Very satisfied

• Somewhat satisfied

• Neither satisfied nor dissatisfied

• Somewhat dissatisfied

• Very dissatisfied

• I didn’t watch the demonstration videos

5. What was your chosen group in the COMiT’ID online survey?

• Member of the public with tinnitus (healthcare user)

• Healthcare practitioner

• Researcher

• Commercial representative/funder

6. What is your country of residence?

*Drop down menu*

7. Is English your first language?

• Yes

• No

8. Which round(s) of the COMiT’ID online survey did you complete? (Please select all that apply)

• Round 1

• Round 2

• Round 3

• I didn't complete any rounds

• Other ______

9. How did you feel about the ease of use of the survey software?

• Very satisfied

• Somewhat satisfied

• Neither satisfied nor dissatisfied

• Somewhat dissatisfied

• Very dissatisfied

10. How did you feel about the length of time between each round of the survey?

• Very satisfied

• Somewhat satisfied

• Neither satisfied nor dissatisfied

• Somewhat dissatisfied

• Very dissatisfied

11. How did you feel about the usability of the 1-9 scoring options?

• Very satisfied

• Somewhat satisfied

• Neither satisfied nor dissatisfied

• Somewhat dissatisfied

• Very dissatisfied

12. How did you feel about the clarity of the descriptions for each outcome domain?

• Very satisfied

• Somewhat satisfied

• Neither satisfied nor dissatisfied

• Somewhat dissatisfied

• Very dissatisfied

13. How did you feel about the usefulness of the option to ‘save and exit’ and then log back into the survey?

• Very satisfied

• Somewhat satisfied

• Neither satisfied nor dissatisfied

• Somewhat dissatisfied

• Very dissatisfied

• I didn't use this option

14. How did you feel about the length of time needed to complete each round of the survey (i.e. round 1, round 2, and round 3)?

• Shorter time than I expected

• Closely matched my expectations

• Longer time than I expected

15. How did you feel about how the study team kept you informed of progress from round to round?

• Very satisfied

• Somewhat satisfied

• Neither satisfied nor dissatisfied

• Somewhat dissatisfied

• Very dissatisfied

16. How did you feel about the frequency of the reminder e-mails from the team?

• Very satisfied

• Somewhat satisfied

• Neither satisfied nor dissatisfied

• Somewhat dissatisfied

• Very dissatisfied

• I did not receive any reminder emails

17. How did you feel about interpreting the graphical display of results in round 3?

• Very satisfied

• Somewhat satisfied

• Neither satisfied nor dissatisfied

• Somewhat dissatisfied

• Very dissatisfied

• I didn't complete Round 3

18. Did your overall experience match what you expected at the start of the study?

• Closely matched my expectations

• Quite different from what I expected

• Prefer not to say.

19. Did you feel about whether your contribution was appreciated?

• Yes

• No

20. Is there anything else that you would like to tell us about?

• ______

Thank you for completing this feedback survey.
