# Supplementary material for: Recruiting and retaining participants in e-Delphi surveys for core outcome set development: Evaluating the COMiT'ID study
Source: PLoS One. 2018 Jul 30;13(7):e0201378. doi: 10.1371/journal.pone.0201378 (PMC6066228; doi:10.1371/journal.pone.0201378)
Supplement: S4 Appendix — List of feedback questionnaire respondents and which country they came from, compared with participants in the e-Delphi surveys. (DOCX) [file pone.0201378.s004.docx]

**S4 Appendix. List of feedback survey respondents and which country they came from, compared with participants in the e-Delphi surveys.**

Countries are grouped according to the World Health Oragnisation regional groupings.

| **Country** | **e-Delphi survey participants** | **Feedback survey respondents** |
| --- | --- | --- |
| **European region** | | |
| Austria | 1 | 1 |
| Belgium | 11 | 7 |
| Bulgaria | 1 | 0 |
| Denmark | 7 | 4 |
| Finland | 1 | 1 |
| France | 31 | 9 |
| Germany | 27 | 19 |
| Greece | 2 | 0 |
| Ireland | 3 | 3 |
| Italy | 10 | 7 |
| Lithuania | 1 | 1 |
| Malta | 1 | 0 |
| Netherlands | 19 | 15 |
| Norway | 2 | 2 |
| Poland | 5 | 2 |
| Portugal | 18 | 6 |
| Romania | 1 | 1 |
| Russia | 1 | 1 |
| Serbia | 1 | 1 |
| Slovenia | 1 | 1 |
| Spain | 10 | 7 |
| Sweden | 5 | 2 |
| Switzerland | 9 | 5 |
| Turkey | 1 | 0 |
| United Kingdom (UK) | 352 | 215 |
| **Northern America** | | |
| United States of America (USA) | 69 | 31 |
| Canada | 15 | 11 |
| **Eastern Mediterranean** | | |
| Iran | 1 | 1 |
| Israel | 2 | 0 |
| Morocco | 1 | 0 |
| **Central and South America** | | |
| Argentina | 2 | 1 |
| Brazil | 9 | 6 |
| Mexico | 1 | 0 |
| **South-east Asia** | | |
| India | 1 | 1 |
| **Western Pacific region** | | |
| Australia | 8 | 6 |
| China | 2 | 0 |
| Malaysia | 2 | 1 |
| Japan | 1 | 0 |
| Singapore | 3 | 1 |
| New Zealand | 2 | 1 |
| **Africa** | | |
| South Africa | 1 | 0 |
| Did not specify | 0 | 9 |
|  | **641** | **379** |
